# Intra-Nasally Administered Oligopeptide Lunasin Acts as a Possible Anti-Psychotic Agent in Mice Models

**DOI:** 10.3390/medicina55070393

**Published:** 2019-07-21

**Authors:** Zane Dzirkale, Ilva Nakurte, Kaspars Jekabsons, Ruta Muceniece, Vija Klusa

**Affiliations:** 1 Faculty of Medicine, University of Latvia; 3 Jelgavas St, LV-1004 Riga, Latvia; 2 Faculty of Chemistry, University of Latvia; 1 Jelgavas St, LV-1004 Riga, Latvia

**Keywords:** lunasin, intranasal administration, hyper-locomotion, brain monoamines, receptor binding

## Abstract

*Background and Objectives:* Previously we have shown that synthetic lunasin, a 43 amino acid residue-containing peptide, after its central (intracisternal) administration in mice demonstrated antagonism against dopaminergic drug behavioural effects, indicating a putative antipsychotic/anti-schizophrenic profile of lunasin. The aims of the present studies were: to test whether lunasin would show an influence on the dopaminergic system after intranasal administration, and to examine the effect(s) of lunasin on serotonin and glutamatergic systems, which could play an essential role in antipsychotic action. *Materials and Methods:* Lunasin was administered intra-nasally at doses 0.1 and 1 nmol/mouse in ICR mice (*n* = 7–8) and tested in an open field on hyperlocomotion caused by amphetamine; serotonin 5-HT 2A/2C receptor agonist 1-(2,5-dimethoxy-4-iodophenyl)- 2-aminopropane (DOI); and glutamate NMDA receptor antagonist phencyclidine. Following behavioural testing, the contents of neurotransmitters and their metabolites in brain hemispheres (*n* = 6–8) were assessed by ultra-high-performance liquid chromatography-time of flight mas-spectrometry (UHPLC-TOF-MS) method. Also, lunasin binding to serotonin receptors was assessed. *Results:* Lunasin intra-nasally fully normalized hyper-locomotion and brain monoamine levels in amphetamine- and DOI-treated mice brains. Phencyclidine behavioural effects were not influenced. In vitro receptor binding data demonstrated a low affinity of lunasin (at µM concentrations) compared with DOI (nM concentrations) for the 5-HT_2A_ and 5-HT_2C_ receptors. *Conclusions:* These results demonstrated, for the first time, that the intranasal administration of oligopeptide lunasin normalized mice behaviour and brain monoamine levels in experimental psychosis mice models. Its neuro-regulatory effects indicated a usefulness of this peptide molecule for the design of novel psychotropic agents.

## 1. Introduction

Peptides are recognized as highly selective, relatively safe, and well tolerated agents. Consequently, there is an increase in peptide pharmacological and pharmaceutical research, and development, particularly focusing on research of novel psychotropic agents [1]. Recent data showed that intra-nasally administered peptides may cross the blood-brain barrier and this type of administration is considered to be the preferred target of the brain [2]. For instance, a variety of peptides and proteins, such as insulin, glucagon-like peptide 1, and neurotrophic factors showed beneficial effects on memory following intranasal administration [3,4]. In the present study, we intra-nasally administered lunasin, a 43 amino acid residue-containing oligopeptide. This peptide was first discovered in soybeans [5], and afterward in cereal grains [6,7] and other plants. During the last two decades, studies performed in cell cultures and animal models have demonstrated the peripheral effects of lunasin, such as anti-cancer and immunomodulatory [8], anti-inflammatory [9,10], antioxidant [11], cholesterol-lowering [12] effects. Our group [13], for the first time, showed that synthetic lunasin, administered centrally (intracisternally) in mice exerted a considerable influence on the central nervous system (CNS) by demonstrating antagonism against dopaminergic drug behavioural effects, and indicating a putative antipsychotic/anti-schizophrenic profile of lunasin.

The aims of the present studies were: (1) to test whether lunasin would show its influence on the dopaminergic system after intranasal administration; (2) to examine the effects of lunasin on serotonin and glutamatergic systems, which could play an essential role in antipsychotic action(s).

We chose three agents selected from the array of dopamine-, serotonin-, and glutamatergic system drugs that induce hyper-locomotion, which has been accepted for modelling positive symptoms of schizophrenia [14]. Thus we treated mice with the dopamine releaser, amphetamine, or serotonin receptor 2A/2C (5-HT_2A/2C_) agonist 1-(2,5-dimethoxy-4-iodophenyl)-2-aminopropane (DOI), or glutamate NMDA receptor antagonist phencyclidine (PCP). After completing the behavioural tests, a neurochemical ex vivo analysis was performed for assessing levels of the brain neurotransmitters: Dopamine and its metabolites homovanillic acid (HVA) and 3,4-dihydroxyphenylacetic acid (DOPAC); noradrenaline; serotonin and its metabolite 5-hydroxyindoleacetic acid (5-HIAA). Ultra-high-performance liquid chromatography-time of flight mass spectrometry (UHPLC-TOF-MS) method was used. Binding studies of lunasin to serotonin 5-HT_2A_ and 5-HT_2C_ receptors were performed in vitro (HEK-293 cells membrane suspensions).

## 2. Materials and Methods

### 2.1. Animals

Male albino ICR (named after the Institute of Cancer Research, Philadelphia, PA, USA) mice were obtained from the Laboratory of Experimental Animals, Riga Stradins University, Latvia. Animals, that weighed 22 ± 2 g, were housed under standard conditions (22–24 °C, 12 h light-dark cycle) with unlimited access to food and water. All experimental procedures were carried out in accordance with the EU Directive 2010/63/EU and local laws and policies on the protection of animals used for scientific purposes, and were approved by the Animal Ethics Committee of the Food and Veterinary Service, Latvia. The experiment was designed in three separate sessions; each one of them consisted of the following experimental groups:

Control 0.9% NaCl solution intraperitoneally (i.p.) + intranasally (i.n.);
0.9% NaCl solution i.p. + lunasin 0.1 nmol/mouse i.n.;
0.9% NaCl solution i.p. + lunasin 1 nmol/mouse i.n.;
Amphetamine 3 mg/kg or DOI 3 mg/kg, or PCP 5 mg/kg i.p. + 0.9% NaCl solution i.n.;
Amphetamine 3 mg/kg or DOI 3 mg/kg, or PCP 5 mg/kg i.p. + lunasin 0.1 or 1 nmol/mouse i.n.

Experimental groups consisted of 6-8 mice each (*n* = 6–8). All efforts were made to minimize animal suffering and to reduce the number of animals used.

### 2.2. Drug Administration

The used reference drugs d-amphetamine at a dose of 3 mg/kg, DOI (1-(2,5-dimethoxy-4- iodophenyl)-2-aminopropane hydrochloride) at a dose of 3 mg/kg, and phencyclidine (1-(1-phenylcyclohexyl)piperidine hydrochloride) at a dose of 5 mg/kg (all from Sigma-Aldrich, USA), were injected in a volume 10 mL/kg i.p. 15 min before intranasal administration of the peptide.

Synthetic lunasin (purchased from CASLO Laboratory ApS, Technical University of Denmark, Denmark) was dissolved in sterile water to prepare a stock solution (1mg/mL) and then diluted with 0.9% NaCl solution to reach the 0.1 and 1 nmole per 5 µL concentrations. The peptide solution at the doses of 0.1 and 1 nmol/mouse/5 µL or 0.9% NaCl solution for control (5 µL/mouse) was administered intra-nasally to conscious and hand-restrained mice, held in a supine position. The solution was applied bilaterally on the rhinarium, the area referred to as the glabrous skin inside the nostrils. The amount of 5 µL was distributed dropwise with the tip of a micropipette, and allowed to diffuse into the nostrils and the squamous epithelium of both the left and right rhinarium.

The influence on locomotion was assessed 15 min after the last intranasal drug administration.

### 2.3. Locomotor Activity

The mouse was placed on the centre of a clear Plexiglas (47 × 36 × 20 cm) open-field arena. Locomotor activity in the open field was quantified by PhenoMaster/LabMaster software (TSE Systems, Germany), which registers the number of light beam interruptions caused by the animal’s movement, and the data are expressed as the total distance travelled in centimetres during the 15-min test.

### 2.4. Sample Preparation and UHPLC-TOF-MS Analysis

At the end of the behavioural test, mice were sacrificed by decapitation and brain hemispheres were removed immediately and stored at −80 °C. The brain hemispheres were weighed and homogenized for 40 s with a homogenizer (T10 basic Ultra Turrax, IKA^®^-Werke GmbH&Co. KG, Germany) in an ice-bath using 750 µL of acetonitrile supplemented with 0.1% formic acid. The obtained homogenate was transferred into an Eppendorf tube. After that, homogenizer was washed with other 750 µL of acetonitrile supplemented with 0.1% formic acid, and the obtained suspension was transferred into the same Eppendorf tube and centrifuged at 13000 **×** g for 15 min. The supernatant was taken for the quantification of biogenic amines and their metabolites in the UHPLC-TOF-MS assay.

Chromatographic analyses were performed on a modular UHPLC system Agilent 1290 Infinity series (Agilent Technologies, Ratingen, Germany). Liquid chromatography (LC) separations were achieved by using an Extend-C18 RRHD (Agilent Technologies, Germany) column 2.1 × 50 mm, 1.8 μm.

Elution solvents consisted of 0.1% formic acid in acetonitrile and 0.1% formic acid in water using a 10-min gradient at a flow rate 0.25 mL/min. The injection volume was 2.0 μL. The high-resolution mass spectra were collected on an Agilent 6230 TOF LC/MS system (Agilent Technologies, Germany) with both positive and negative electrospray ionization. The source parameters were as follows: drying gas flow 12.0 l/min, temperature 325 °C, fragmentor ionization 130 V. One full mass spectrum was acquired in profile mode, with a mass range from m/z 115 to 250. The internal reference mass of 121.050873 m/z (G1969-85001 ES-TOF Reference Mass Solution Kit, Agilent Technologies, Germany) was used for all analyses of the samples. The experimental data were handled using MassHunter version B05.0 software (Agilent Technologies, Germany).

Dopamine, noradrenaline, serotonin, HVA, DOPAC, and 5-HIAA were purchased from Sigma-Aldrich. The purity of these reference analytical standards was more than 98%. The identification of separated dopamine, noradrenaline, and serotonin were based on the search for [M+H]^+^ ions, while identification of HVA and 5-HIAA were based on the search for [M-H]^-^ ions and DOPAC for [M-CO_2_-H]^-^, using extracted ion mass chromatograms and taking into account the data provided by the external standard. The experimentally obtained mass spectra of all compounds approved the calculated values: NA (C_10_H_12_N_2_O, (M-H_2_O+H)^+^ Calculated – 152.0715, Found – 152.0712, Δ 0.0003), DA (C_8_H_11_NO_2_, (M+H)^+^ Calculated – 154.0863, Found – 154.0869, Δ 0.0006) 5-HT (C_10_H_12_N_2_O, (M+H)^+^ Calculated – 177.1022, Found – 177.1028, Δ 0.0006); HVA (C_9_H_10_O_4_, (M-H)^-^ Calculated – 181.0506, Found – 181.0514, Δ 0.0008); 5-HIAA (C_10_H_9_NO_3_, (M-H)^-^ Calculated – 190.0510, Found – 190.0519, Δ 0.0009); DOPAC (C_10_H_9_NO_3_, (M-CO_2_-H)^-^ Calculated – 123.0000, Found – 123.0016, Δ 0.0016). The calibration curve of each standard solution was constructed by plotting the ratio of the average chromatographic peak area and mass concentration. According to the reflected data, the regression equation of the trend line was calculated. Standard solutions were injected in triplicate, and the corresponding peak areas were recorded. Stock solutions of dopamine, HVA, DOPAC, noradrenaline, serotonin, and 5-HIAA at a concentration of 1 µg/mL were prepared in 50% acetonitrile in water. Working solutions of all the standards were prepared immediately before analyses by diluting the stock solution with water to attain the required concentrations for calibration measurements. The relative standard deviation was determined to be less than 2.0%. The obtained calibration curves showed linearity of the correlation coefficient (r^2^) in the concentration range of 0.9991–0.9998. The coefficient of determination (r^2^) was calculated using Microsoft Excel 2013 (Microsoft, Redmond, WA, USA), *p* < 0.001.

### 2.5. Binding Assays

The binding assay was performed by Eurofins Cerep (France) according to Bryant et al., 1996 [15]. Briefly, the binding of lunasin to 5-HT_2A_ and 5-HT_2C_ receptors was estimated on its ability to displace the specific binding of [^125^I]DOI on cell membrane suspensions of HEK-293 cells expressing human recombinant 5-HT_2A_ and 5-HT_2C_ receptors. The radioligand [^125^I]DOI has high affinity for 5-HT_2A_ (K_d_: 0.3 nM) and 5-HT_2C_ (K_d_: 0.9 nM) receptors. Lunasin was tested at concentrations of 1, 10 and 100 µM. The results were expressed as a percent of radioligand specific binding (*n* = 2).

### 2.6. Statistics

All the experimental data were analyzed with GraphPad Prism 6 software (GraphPad Software Inc., CA, USA) using one-way ANOVA with Fisher’s LSD test as post hoc analysis. The results are expressed as the mean ± SD. A significance level was set at *p* < 0.05.

## 3. Results

### 3.1. Influence on Locomotion

**Figure 1:**
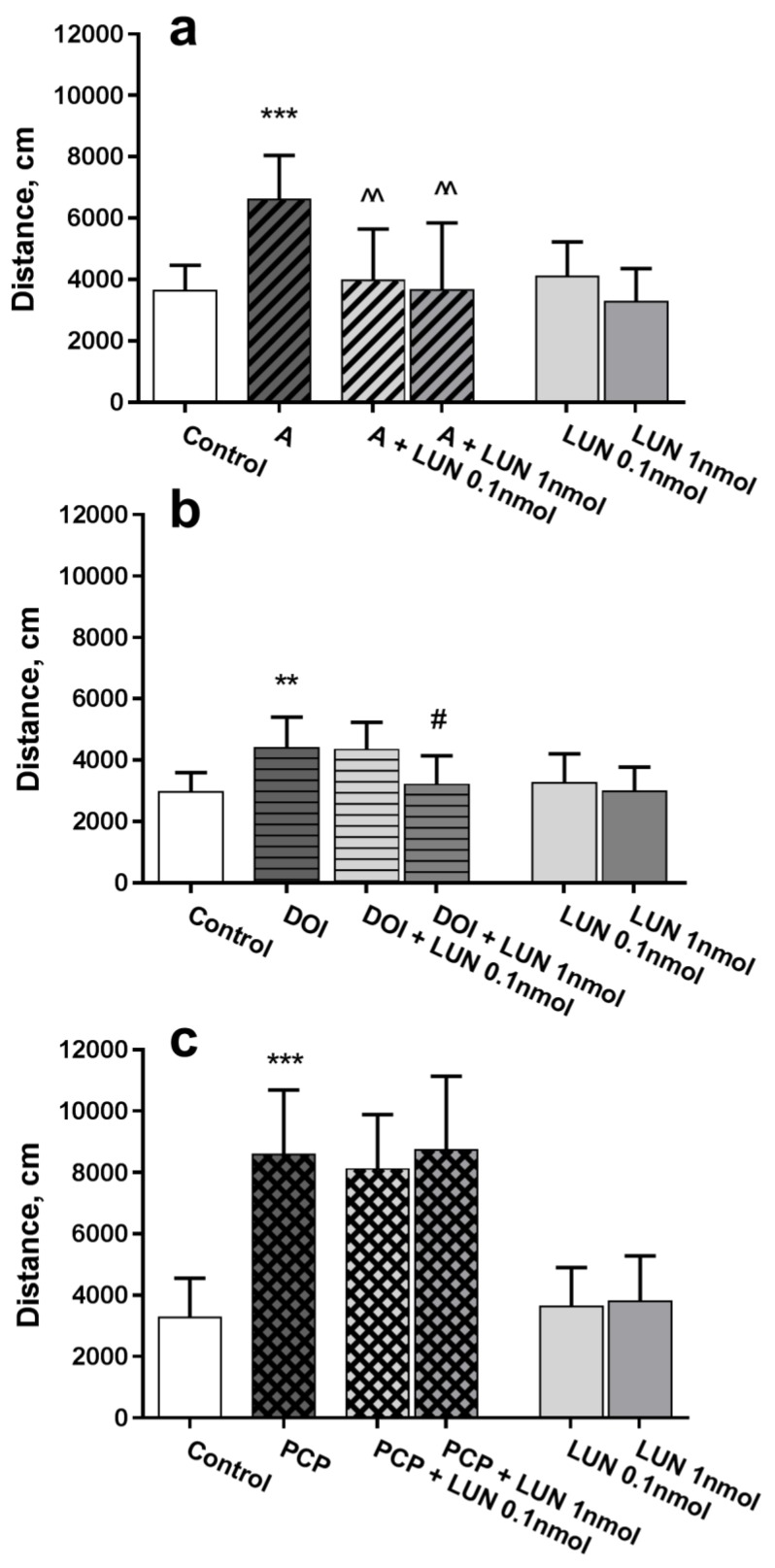
Influence of lunasin i.n. on locomotor activity in an open field test in ICR mice. Drugs that produce hyperactivity, such as amphetamine (A) 3 mg/kg (**a**); DOI 3 mg/kg (**b**); and phencyclidine (PCP) 5 mg/kg (**c**) were administered i.p. 15 min before lunasin (LUN) doses at 0.1 or 1 nmol/mouse/5 µL or 0.9% NaCl i.n. (Control). Locomotor activity was assessed 15 min after i.n. administration and quantified as the distance travelled in cm during the 15-min test. Data are expressed as the mean ± SD, (*n* = 7–8). ** *p* < 0.01 and *** *p* < 0.001 vs. Control; ^^ *p* < 0.01 vs. A (a); # *p* < 0.05 vs. DOI (b).

In the open field test, amphetamine caused a considerable, approximately 2-fold, increase in total walked distance versus the control (*p =* 0.0008), whereas lunasin, at the both tested doses 0.1 and 1 nmol/mouse, administered intranasally, considerably decreased (*p =* 0.0031, *p =* 0.0018, respectively) the hyperlocomotion (Figure 1a). Similarly, the 5-HT_2A_ and 5-HT_2C_ receptor agonist DOI induced hyperactivity versus the control (*p =* 0.0033); lunasin administration at a dose of 1 nmol/mouse decreased hyperactivity (*p =* 0.0137, Figure 1b). The NMDA receptor antagonist PCP induced hyperlocomotion, which differed substantially versus the control (*p* < 0.0001); however, lunasin did not influence the PCP effect (Figure 1c). Intranasally administered lunasin per se did not influence mouse activity; the walked distance during the 15-min test was about the same as for the control group (Figure 1).

### 3.2. Neurochemical Data

**Figure 2:**
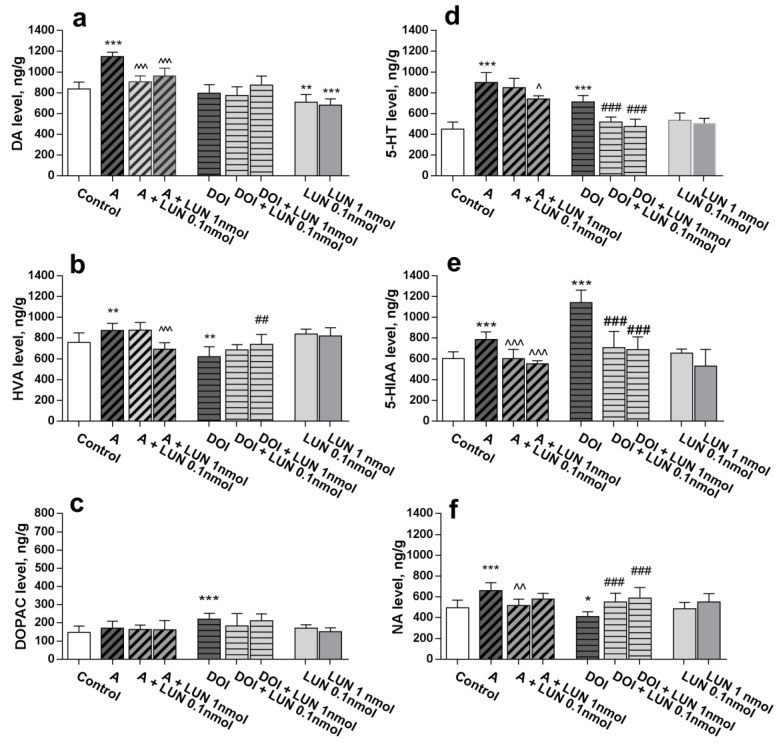
Influence of lunasin i.n. on brain monoamines and their metabolite levels altered by amphetamine (A) and DOI: DA (**a**), HVA (**b**), DOPAC (**c**), 5-HT (**d**), 5-HIAA (**e**), and NA (**f**) levels (ng/g). Data are expressed as the mean ± SD, *n* = 6–8. * *p* < 0.05, ** *p* < 0.01 and *** *p* < 0.001 vs. Control; ^ *p* < 0.05, ^^ *p* < 0.01, *p* < 0.001 vs. A; ## *p* < 0.01, ### *p* < 0.001 vs. DOI.

The neurochemical data demonstrated that lunasin i.n. at tested doses, 0.1 and 1 nmol/mouse, influenced the monoamine levels in the amphetamine-treated mouse brains. Amphetamine markedly increased the dopamine level (*p* < 0.0001), which was reversed to control values by lunasin treatment (*p* < 0.0001). Additionally, lunasin significantly decreased dopamine levels (*p* < 0.01, Figure 2a). DOI did not change dopamine brain concentrations (Figure 2a). The dopamine metabolite HVA level was increased by amphetamine (*p =* 0.0079) but decreased by DOI treatment (*p =* 0.0022); lunasin at 1 nmol/mouse normalized the altered levels of HVA (*p* < 0.001, and *p* < 0.01, respectively, Figure 2b). Neither lunasin per se nor amphetamine changed the DOPAC concentration; it was elevated (*p* < 0.001) only by DOI (Figure 2c). The noradrenaline level was augmented by amphetamine (*p* < 0.0001) and decreased by DOI treatment (*p* < 0.05). Lunasin 0.1 nmol/mouse reversed amphetamine-induced and DOI-induced (both tested doses) noradrenaline alterations to the control values (*p* < 0.01, Figure 2f). Treatment with amphetamine increased brain serotonin and its metabolite 5-HIAA levels (*p* < 0.0001), while lunasin 1 nmol/mouse decreased the augmented serotonin (*p* < 0.01, Figure 2d) at both doses, as well as 5-HIAA levels (*p* < 0.0001, Figure 2e). Similarly, lunasin reversed the DOI up-regulated levels of serotonin and 5-HIAA almost to the control values (*p* < 0.0001, Figure 2d, Figure 2e).

Summarizing data from Figure 1 and Figure 2 are shown in Table 1.

### 3.3. Lunasin Binding to 5-HT_2A_ and 5-HT_2C_ Receptors

Lunasin at the concentrations of 1 and 10 µM did not show binding activity, whereas at 100 µM it demonstrated 59.7% inhibition of DOI specific binding to the 5-HT_2C_ receptor. However, for the 5-HT_2A_ receptor, lunasin at only the 100 µM concentration showed 32.5% inhibition of DOI-specific binding (Table 2).

## 4. Discussion

Our previous study demonstrated that after direct central administration (intracisternally) lunasin reduced amphetamine-induced hyper-locomotion and apomorphine-induced climbing activity. The peptide bound to the dopamine D_1_ receptor subtype, has an affinity similar to that of dopamine, but does not bind to the dopamine D_2_ receptor subtype [13]. The present study showed for the first time that synthetic lunasin (doses 0.1 and 1 nmol) was also active after intranasal administration in mice. Thus, lunasin at both doses normalized to control the level of behavioural changes after amphetamine injection. This similarity of effects indicate that lunasin could reach the CNS after intranasal administration. In this context, our findings are in good agreement with previously reported data that natural lunasin isolated from plants may reach the brain after oral administration [16]. The neurochemical data (UHPLC-TOF-MS method) obtained in the brains of amphetamine-treated mice confirmed the lunasin regulatory effects on the dopaminergic system, since it reversed to control value the elevated levels of dopamine and its metabolite HVA (at 1 nmol/mouse). Another dopamine metabolite DOPAC was not affected either by lunasin or by amphetamine. Lunasin per se significantly decreased dopamine level. At the dose 0.1 nmol/mouse, it also normalized noradrenaline level to control values in amphetamine-treated mice.

Intriguing are the data that lunasin 1 nmol/mouse fully normalized the levels of serotonin and its metabolite 5-HIAA (both tested doses), which were increased by amphetamine. That can be explained by data suggesting the influence of amphetamine on adrenergic and serotoninergic systems by increasing the cellular interactions among dopamine, serotonin, adrenaline, opiates, histamine, and other signalling molecules, probably via monoamine transporter proteins [17].

Looking at lunasin as a potential antipsychotic agent, particularly important are the present data demonstrating that lunasin might normalize behaviour altered by DOI, a ligand that is related to both 5-HT_2A_ and 5-HT_2C_ receptors [18]. An important role of 5-HT_2A_ and 5-HT_2C_ receptors was shown for the treatment of schizophrenia. Now it is accepted that mostly used atypical antipsychotic drugs (e.g., quetiapine), which involve serotoninergic components may reduce the debilitating side effects, e.g., extrapyramidal manifestations, caused by dopamine receptor antagonistic action. Although both subtype systems have a functional antagonism, even at the same neurons [19], this phenomenon manifests as different effects on locomotion, cognition, and other behavioural events [20]. These receptors are also linked to hallucinogen activity [21], and serve as a therapeutic target for drug abuse and addiction [22]. Moreover, many other 5-HT receptor subtypes, such as 5-HT_1A_, 5-HT_3_, 5-HT_6_, and 5-HT_7_ receptors are also involved in the atypical drug antipsychotic effects [23].

The selected DOI dose in our behavioural studies was 3 mg/kg, which according to published data [24], corresponds to the dose range (0.625-5.0 mg/kg) that induces hyper-locomotion. Lunasin (1 nmol/mouse) exerted protection against DOI-induced hyper-locomotion. Neuro-chemically, DOI caused a considerable elevation of brain levels of serotonin and its metabolite 5-HIAA, while lunasin (0.1 and 1 nmol/mouse) fully normalized them to the control values. DOI did not change the dopamine concentration but lowered those of HVA and noradrenaline, while increasing DOPAC levels. These altered parameters were normalized by lunasin. A cross-talk between 5-HT_2A_ and D_2_ receptors is also found elsewhere [25].

The present study showed weak binding activity of lunasin (at 100 µM) to 5-HT_2A_ and more considerable to 5-HT_2C_ receptors (Table 2). The data obtained in the DOI test, similarly to those in the amphetamine test, confirmed the regulatory profile of lunasin, suggesting that the normalizing effects of the serotonin and its metabolite levels are apparently much more significant than the binding activity.

The complexity of 5-HT receptor multi-functionality involves, not only interactions between serotonin and dopamine, but also between serotonin and glutamate signalling [26]. Therefore, it was important to test whether lunasin may affect the glutamatergic system, which is considered as an essential component in schizophrenic behaviour. In the present study PCP, a glutamate (NMDA) receptor antagonist, was used. However, we did not find any influence of lunasin on PCP-induced hyperactivity. That concurred with our previous data that lunasin central (intracisternal) injection did not influence locomotion altered by ketamine – another NMDA receptor antagonist [13]. Therefore, the glutamatergic system is likely not a key player in lunasin’s central action and, thus, we did not assess brain monoamine levels for PCP-treated mice.

## 5. Conclusions

In summary, the present findings for the first time demonstrated the strong normalizing action of intra-nasally administered lunasin, by reversing the behaviour and brain monoamine levels to control parameters altered by amphetamine and DOI, indicating possible antipsychotic efficacy of this peptide. Regulating action may be considered as a priority in comparison to routine antipsychotic drugs affecting the certain neurotransmitter receptor type or subtype, thus leading to different adaptation detrimental responses (side effects) in other systems. Moreover, the efficacy of lunasin intranasal administration broadens the applicability of this oligopeptide for therapeutics. Certainly, much remains to be clarified in relation to the the influence of lunasin on nasal mucosa after long-term intranasal administration, and further tests are requires to understand its effects in other animal models related to psycho-neurological diseases.

## Figures and Tables

**Table 1:** Summarizing data: Influence of intranasally administered lunasin (0.1 and 1 nmol/mouse) on locomotor activity (distance, cm) and brain monoamine levels (ng/g), changed by amphetamine (3 mg/kg i.p.) and DOI (3 mg/kg i.p.).

Compounds	Locomotion	DA	HVA	DOPAC	5-HT	5-HIAA	NA
**Amphetamine**	↑	↑	↑	Nc	↑	↑	↑
**Amphetamine + Lunasin 0.1**	N	N	Nc	Nc	Nc	N	N
**Amphetamine + Lunasin 1**	N	N	N	Nc	N	N	Nc
**DOI**	↑	Nc	↓	↑	↑	↑	↓
**DOI + Lunasin 0.1**	Nc	Nc	Nc	Nc	N	N	N
**DOI + Lunasin 1**	N	Nc	N	Nc	N	N	N
**Lunasin 0.1**	Nc	↓	Nc	Nc	Nc	Nc	Nc
**Lunasin 1**	Nc	↓	Nc	Nc	Nc	Nc	Nc

↑—increase; ↓—decrease; N—normalization; Nc—no changes.

**Table 2:** Lunasin binding to 5-HT_2A_ and 5-HT_2C_ receptors: inhibition of [^125^I]DOI specific binding (%), data are expressed as the mean of duplicates.

Concentration/Receptor	5-HT_2A_	5-HT_2C_
**1 µM**	1.6%	4.7%
**10 µM**	2.6%	10.3%
**100 µM**	32.5%	59.7%
